# Desensitizing toothpastes for dentin sealing and tertiary dentin formation in vitro and in vivo: a comparative analysis

**DOI:** 10.1186/s12903-022-02558-8

**Published:** 2022-11-11

**Authors:** Juhyun Lee, Geumbit Hwang, Hyeri Gug, Ji-Hyun Lee, Su-Jin Park, Joo-Cheol Park

**Affiliations:** 1 Department of Oral Histology-Developmental Biology & Dental Research Institute, Laboratory for the Study of Regenerative Dental Medicine, School of Dentistry, Seoul National University, 1 Gwanak-ro, Gwanak-gu, Seoul, 08826 Republic of Korea; 2 HysensBio, Co., Ltd., 10 Dwitgol-ro, Gwacheon-si, Gyeonggi-do Republic of Korea

**Keywords:** Dentin hypersensitivity, Desensitizing toothpaste, CPNE7-DP, Dentin sealing, Tertiary dentin

## Abstract

**Background:**

Dentin hypersensitivity is a painful response to external stimuli applied to exposed dentinal tubules. Various toothpastes with active desensitizing ingredients for the relief of dentin hypersensitivity are commercially available. However, data from several studies suggest that the effects of desensitizing toothpastes are unstable and brief. This study aimed to investigate the effect of toothpastes containing CPNE7-derived oligopeptide (CPNE7-DP) and other active desensitizing ingredients in the dentin microleakage, tubule occlusion and tertiary dentin formation.

**Methods:**

Using scanning electron microscopy (SEM), we evaluated the patency of dentinal tubules on the surface of human dentin disks after brushing experiments with the various toothpastes. Dentin was histologically evaluated in a hypersensitivity model of canine teeth, after the exposed dentin area was brushed for 6 weeks. The toothpaste used in group 1 (control) did not contain any desensitizing ingredients; that used in group 2 contained CPNE7-DP; Colgate Sensitive was used in group 3; and Sensodyne Rapid Relief was used in group 4. Finally, we conducted microleakage analysis to investigate the dentin sealing effect. The microleakage analysis data were subjected to one-way ANOVA and post-hoc Tukey tests (alpha = 0.05).

**Results:**

In the SEM images, all four groups of teeth exhibited partial occlusion of the dentinal tubules on the tooth surface. In the in vivo hypersensitivity model, group 2 exhibited a newly formed tertiary dentin, whereas no new hard tissue formation was observed in groups 1, 3, and 4. Microleakage analysis revealed that the volume of dentinal fluid flow was significantly smaller in group 2 than in group 1.

**Conclusions:**

These results indicate that CPNE7-DP is a promising active ingredient with long-term dentin sealing effects.

**Supplementary Information:**

The online version contains supplementary material available at 10.1186/s12903-022-02558-8.

## Background

Dentin hypersensitivity is a prevalent problem in dental practice and affects approximately 25% of the adult population [1, 2]. It is described as brief but sharp pain that arises from exposed dentin in response to external stimuli and that cannot be attributed to any other form of dental disease or defect [3]. Among the explanations for these painful response, the hydrodynamic theory is generally accepted [4]. According to this theory, the cause of dentin hypersensitivity is the increased fluid movement within open dentinal tubules [5]. The painful symptoms of dentin hypersensitivity can be reduced by dentinal tubule occlusion [5, 6].

Desensitizing toothpaste—a dental product used at home—is the first choice for dentin hypersensitivity owing to its convenience, low cost, and noninvasiveness [7, 8]. Various desensitizing toothpastes are commercially available. These toothpastes exert their effect by blocking pulpal nerve responses or occluding dentinal tubules [9]. Toothpastes for blocking pulpal nerve response contain potassium salts; however, the efficacy of potassium salts is controversial [9, 10]. The majority of desensitizing toothpastes work by occluding dentinal tubules and contain several active ingredients, including calcium carbonate, arginine, and strontium acetate [9, 11]. Previous studies have demonstrated that these active ingredients act as desensitizing agents by reducing pain [12–16]. Although tubule occlusion was observed in several studies, it was superficial and not resistant to acid challenges and saliva immersion [17, 18]. Therefore, desensitizing agents with long-term tubule-occluding effects must be developed using appropriate assessment methods.

The copine family comprises ubiquitous calcium-dependent, phospholipid-binding proteins that are highly conserved across several species [19]. One copine, CPNE7, reactivates odontoblasts and promotes the formation of physiological dentin [20] and reacts with calcium ion in dentinal fluid owing to its high calcium ion-binding affinity [21]. In a dentin hypersensitivity model, CPNE7 induced biological dentin sealing, and the effect was permanent rather than transient [22]. Subsequently, Lee et al. synthesized CPNE7-derived oligopeptide (CPNE7-DP), which, as recombinant CPNE7 protein, also induced dentinal tubule occlusion [23].

Therefore, this study aimed to evaluate the effect of CPNE7-DP–containing toothpaste and two commercial desensitizing toothpastes on dentinal tubule occlusion and sealing. Tubule occlusion on the dentin surface was evaluated via scanning electron microscopy (SEM). To investigate the long-term dentin sealing effect in clinical situations, tertiary dentin formation was analyzed in an in vivo hypersensitivity model after the teeth were brushed with the toothpastes. Dentin permeability and sealing were measured using a microleakage measuring device.

## Methods

### Human dentin preparation

The experimental protocol for this study was approved by the Institutional Review Board in Seoul National University Dental Hospital, Seoul, Korea (S-D20140007). The experiments involving extracted human teeth were performed in accordance with the Declaration of Helsinki. Informed written consent was obtained from each participant prior to the experiments. Five extracted human molars were obtained from Seoul National University Dental Hospital, stored in phosphate-buffered saline (PBS) at 4 °C before use, and then sectioned mid-coronally into 1-mm-thick dentin disks. Each disk was sectioned into four fan-shaped pieces by a low-speed diamond wheel saw (Model 650; South Bay Technology Inc., San Clemente, CA, USA) under constant water cooling. The pieces were rinsed with PBS twice for 5 min each and immersed in 0.5-M ethylenediaminetetraacetic acid (EDTA) solution for 5 min to remove inorganic debris. The processed pieces were then washed again with PBS twice for 5 min each. To remove the smear layer and fully open the dentinal tubules, the pieces were etched with 32% phosphoric acid for 5 min and sonicated by an ultrasonic processor (VCX-750; Sonics & Materials, Inc., Newtown, CT, USA) six times for 5 min each. The pieces were washed with 1X PBS three times for 5 min each and then stored in artificial saliva. The artificial saliva consisted of 0.7-mM CaCl_2_, 30-mM KCl, 0.2-mM MgCl_2_•6H_2_O, 4.0-mM NaH_2_PO_4_, 0.3-mM NaN_3_, and 20-mM HEPES buffer [24].

### In vitro treatment of human teeth

From the five human teeth, 20 dentin disks were randomly divided into five groups (*n* = 4). The disks in the negative control group were kept in artificial saliva and were not brushed at all. The disks in the other four groups were brushed with different toothpastes: group 1 (control), with toothpaste not containing any desensitizing ingredients; group 2, with toothpaste containing CPNE7-DP (HysensBio Co., Ltd., Gwacheon, Korea); group 3, with Colgate Sensitive Complete Protection Toothpaste (Colgate-Palmolive Company, New York, NY, USA); and group 4, with Sensodyne Rapid Relief (GlaxoSmithKline, Brentford, UK). The toothpastes had different active ingredients, which are summarized in Table 1.

**Table 1:** Summary of the experimental groups

Group	Product name	Active ingredient	Company
1	Control (non–desensitizing ingredients)	–	
2	CPNE7-DP–containing toothpaste	CPNE7-DP	HysensBio Co.
3	Colgate Sensitive Complete Protection Toothpaste	Calcium carbonate, arginine	Colgate-Palmolive Company
4	Sensodyne Rapid Relief	Strontium acetate	GlaxoSmithKline

*CPNE7-DP* CPNE7-derived peptide

Each group was divided into two subgroups characterized by the duration of toothpaste application (2 weeks or 4 weeks); each subgroup comprised two dentin disks. The toothpastes were applied to the disks with microbrushes (M6500-F Micro Applicator; TPC Advanced Technology Inc., City of Industry, CA, USA), and each disk was manually brushed for 1 min twice a day for 2 or 4 weeks. After each brushing session, the disks were washed with distilled water and immersed in artificial saliva until the next brushing session.

### Preparation of experimental toothpastes

The toothpastes of Group 1 (Control) and Group 2 (CPNE7-DP—containing toothpaste) were manufactured in the lab, excluding the two commercial toothpastes. The toothpaste of Group 2 was the addition of CPNE7-DP to the toothpaste of Group 1, and all other components are the same. CPNE7-DP was synthesized as mentioned in the previous study [23]. For experimental toothpaste production, first, purified water and D-sorbitol solution were mixed. Second, Tricalcium phosphate, aminocaproic acid, allantoin, hydrous silicic acid, sodium PCA solution, hydroxyapatite, CPNE7-DP (Group 2 only), enzyme-treated stevia, and xylitol were added and stirred in a stirrer for about 40 minutes. Third, (concentrated) glycerin and xanthan gum were added and stirred for about 40 minutes. Fourth, (concentrated) Glycerin, Carboxymethyl Cellulose Sodium Salt (CMC) were added and stirred for about 40 minutes. Stirring conditions from steps 2 to 4 were as follows; PADDLE 10-30 rpm, DISPERSE 500-600 rpm, HOMO 2400-3200 rpm. Fifth, sodium cocoylmethyltaurate were added and stirred for about 20 minutes. Finally, the Flavoring agents were added and stirred for about 15 minutes. The stirring conditions for steps 5 and 6 were PADDLE 10-30 rpm, DISPERSE 450-650 rpm. Each step is stirred under reduced pressure conditions (− 760 mmHg). The detailed ingredients and contents of toothpaste are recorded in supplementary Table 1.

### SEM analysis

The disks brushed with toothpaste were fixed in 0.1 M of cacodylate buffer (pH 7.4) containing 2.5% glutaraldehyde for 30 min and in 0.1 M of cacodylate buffer containing 1% osmium tetroxide for 1 h. The disks were dehydrated in graded acetone and then critical point dried. Each sample was sputter-coated with a thin layer of gold and observed under the scanning electron microscope (S-4700; Hitachi, Ltd., Tokyo, Japan) at an accelerating voltage of 10 kV.

### In vivo dentin hypersensitivity model with canine teeth

All experiments involving animals followed the protocols approved by the Ethics and Institutional Animal Care and Use Committee of Seoul National University (SNU-180416-2-1 and SNU-171020-5-2). This study also conformed to the Animal Research: Reporting In Vivo Experiments (ARRIVE) guidelines for preclinical animal studies. Four beagles (aged between 1 and 2 years) were obtained from the Experimental Animal Center of College of Dentistry and Use Committee of Seoul National University and used for the in vivo dentin hypersensitivity model. Each group consisted of 1 beagle dog with 10 premolars (2 maxillary premolars and 3 mandibular premolars on each side). Before the preparation of the cervical area on the buccal side, all the calculus was removed. For disinfection before dentin exposure, the tooth surfaces were swabbed with cotton balls soaked in 0.5% chlorhexidine. To mimic class V cervical lesions in human patients with dentin hypersensitivity, a high-speed handpiece was used to create modified class V – like cavities on the buccal surfaces of the teeth. The depth of the cavities was half the diameter of the high-speed round bur (Carbide Bur FG Round #4, FG4-014; Komet Dental, Lemgo, Germany). The smear layer was removed with 17% EDTA for 2 min. All defects were exposed during the tooth brushing period. All toothpastes were applied using the Bass brushing method, 1.5 min for each quadrant, once daily for 6 weeks. The samples were obtained after vital perfusion with the Karnovsky solution.

### Histological analysis

The premolars (*n* = 5) were extracted, fixed in 4% paraformaldehyde, decalcified in 30% formic acid, and embedded in paraffin. The samples were coronally divided, perpendicular to the cavity, into 5-μm-thick sections. The sections were stained with hematoxylin and eosin and observed under an optical microscope (Axiolab 5; ZEISS Microscopy, Jena, Germany).

### Microleakage analysis

To evaluate the microleakage of dentinal fluid from the cervical lesions, the apical 3 mm of single roots was eliminated (*n* = 5). A high-speed carbide bur (FG#330; SS White Dental, Seoul, Korea) was used to prepare each root end for a 2-mm depth along the root canal, and a 0.9-mm metal tube was inserted into the canal. To insert the metal tubes, we used 37% phosphoric acid (Any-Etch™; Mediclus Co., Ltd., Cheongju, Korea), adhesive agent (3 M™ Single Bond Universal Adhesive; 3 M ESPE, St. Paul, MN, USA), and flowable composite resin (3 M™ Filtek™ Supreme Ultra Flowable Restorative; 3 M, Alexandria, MN, USA). The whole surfaces of the teeth were coated with nail polish several times, except the defect area. The prepared samples were kept in distilled water until the microleakage test. The microleakage test was conducted as previously described [22]: a machine (nanoFlow; IB Systems, Seoul, Korea) was used to measure the movement of the bubbles (indicating leakage) caused by the flow of distilled water from the tooth apex to the exposed dentin at 70-cm H_2_O. All measurements were taken at 40 min after connecting the sample. The 20-min outflow was recorded, excluding the initial 20-min outflow.

### Statistical analysis

Statistical analyses were performed using GraphPad Prism software (version 5, GraphPad Software, CA, USA). All values are expressed as the mean ± standard deviation for at least three independent experiments. The normal distribution was confirmed using the Kolmogorov-Smirnov test with Dallal-Wilkinson-Lillie for *p*-value (alpha = 0.05). Between-group statistical analyses were performed using the one-way analysis of variance followed by Tukey’s multiple comparison test (p-value < 0.05). Statistically significant differences between groups were considered at *p < 0.05, **p < 0.01 and ***p < 0.001.

## Results

### SEM analysis of dentinal tubule

**Fig. 1:**
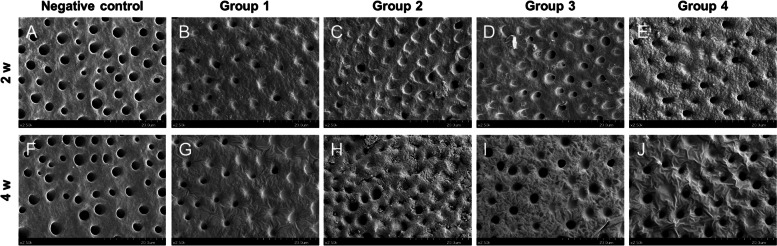
Scanning electron microscopic images of human dentin disks at 2500x magnification, after brushing for 2 or 4 weeks. The upper row presents the disks after 2 weeks of toothpaste application, and the lower row presents those after 4 weeks of application. In the negative controls (**A** and **F**), the dentin surface appeared free of smear layer, and the dentinal tubules were completely open. In the experimental disks (**B**–**E** and **G**–**J**), partial occlusion of dentinal tubules was observed

After the 2- or 4-week protocol of tooth brushing, the surface images of the dentin disks were analyzed via SEM at 2500x magnification. The negative control disks showed that the smear layer was removed, and dentinal tubules were fully open (Fig. 1A, F); on the contrary, all experimental disks revealed partially occluded dentinal tubules (Fig. 1B–E, G–J). In groups 2 and 3, the disks brushed for 2 weeks exhibited more tubule occlusion than did those in groups 1 and 4 (Fig. 1C, D). Among all experimental groups, the disks brushed for 4 weeks exhibited a slightly heavier deposition on the intertubular dentin and firm occlusion of the dentinal tubules, in comparison with those brushed for 2 weeks. However, we found few other differences in each group between the disks brushed for 2 weeks and those brushed for 4 weeks. These results indicate that toothpastes cause partial occlusion of open dentinal tubules.

### Tertiary dentin formation at the dentin–pulp interface

**Fig. 2:**
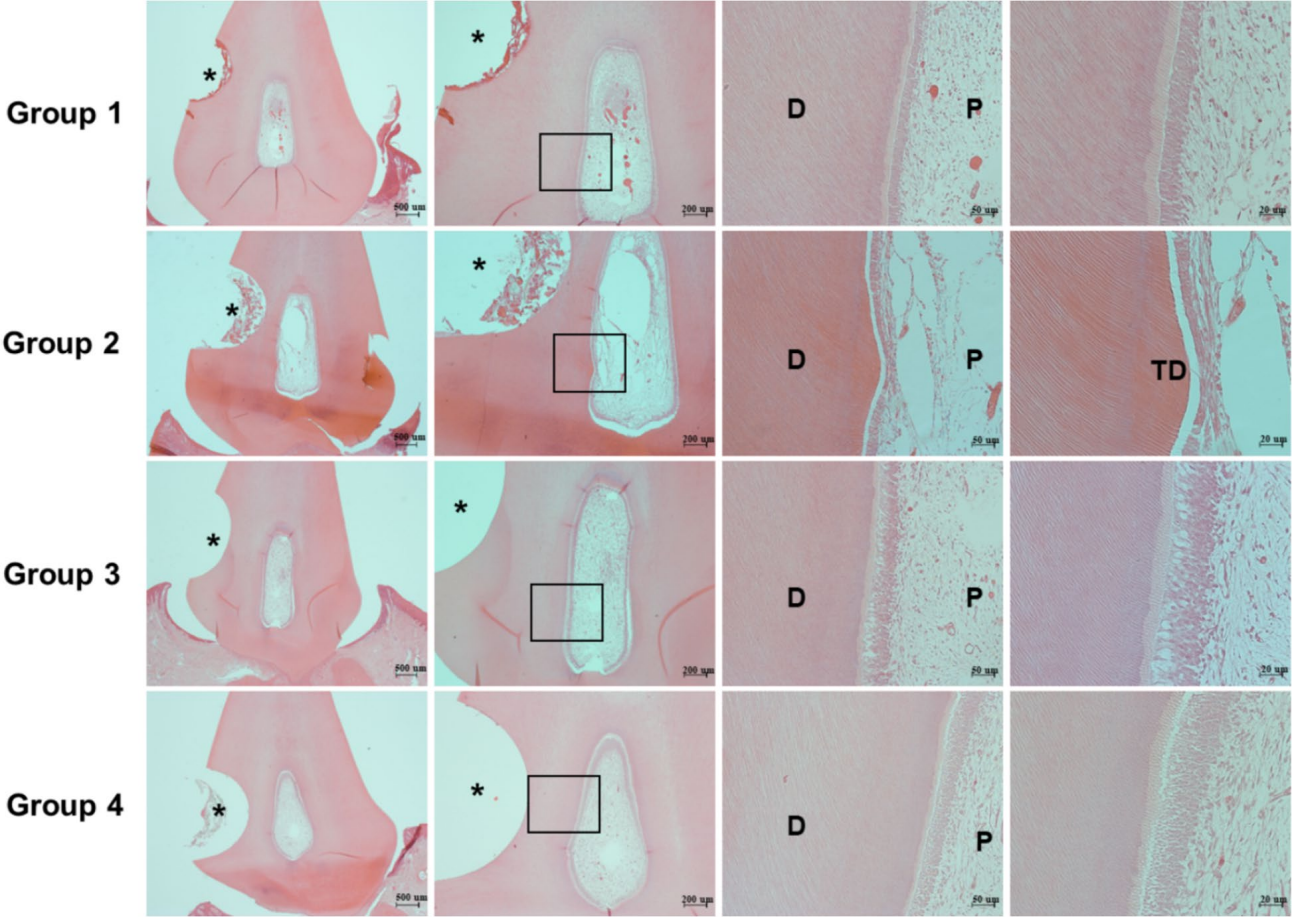
Histologic analysis after application of various toothpastes in the dentin hypersensitivity model with canine teeth. The toothpastes were applied using the Bass method to all groups of teeth, once daily for 6 weeks. In groups 1, 3, and 4, no histologic changes were observed in the dentin–pulp interface. In contrast, group 2 showed newly formed tertiary dentin. Boxed areas are shown at higher magnification. The asterisk indicates the defect area; D represents dentin; P represents pulp; TD represents newly formed tertiary dentin

To evaluate long-term dentin sealing effects, we generated an in vivo hypersensitivity model with dogs’ teeth. After the exposed dentin area was brushed for 6 weeks, we histologically analyzed the dentin. As presented in Fig. 2, no new mineralized tissue formation was observed in groups 1, 3, and 4. Conversely, group 2 demonstrated newly formed tertiary dentin underneath the tooth defect area; moreover, this newly formed dentin included dentinal tubules. These findings indicate that only the CPNE7-DP–containing toothpaste caused biological tertiary dentin to form, which could result in a long-term dentin sealing effect.

### Comparison of permeability and sealing ability

**Fig. 3:**
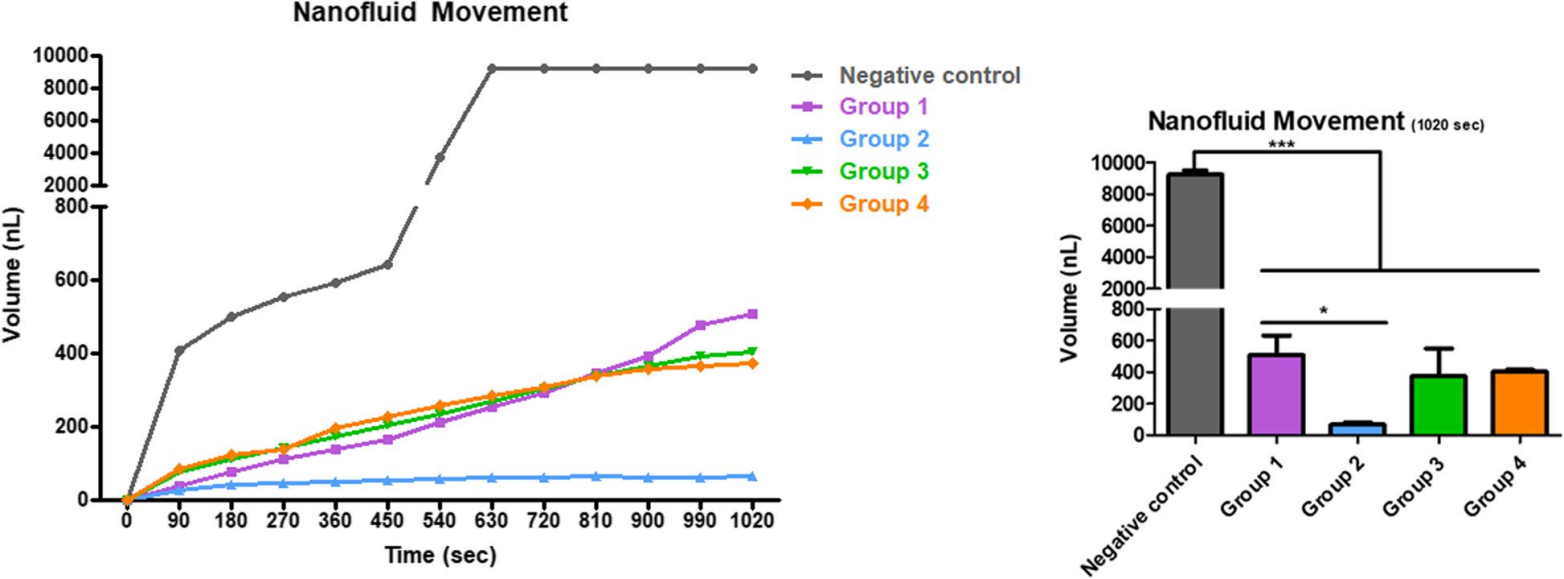
Representative graphs of dentinal fluid flow after application of various toothpastes. This flow progressively increased in groups 1, 3, and 4; that of group 2 was significantly lower than that of the group 1. (*p < 0.05 and ***p < 0.001)

The representative graphs of dentinal fluid flow according to time are presented in Fig. 3. The negative control demonstrated a rapid increase in dentinal fluid flow over time because the dentinal tubules were fully open. In groups 1, 3, and 4, the dentinal fluid flow progressively increased because the dentinal tubules were partially occluded. Subsequently, a statistical analysis was performed using the volume of the dentinal fluid flow at 1020 sec. In comparison to the negative control, all groups presented noticeably reduced dentinal fluid flow. The volume of dentinal fluid flow was significantly smaller in group 2 than in group 1, although the dentinal tubules were only partially blocked, according to the SEM images of the dentin surface. On the other hand, the volume of dentinal fluid flow in group 3 and 4 did not differ significantly compared to that of group 1. These results indicate that the CPNE7-DP–containing toothpaste had a better dentin sealing effect than did the toothpaste of group 1.

## Discussion

Exposure of dentin caused by gingival recession or tooth abrasion is a major cause of dentin hypersensitivity [25]. When various stimuli (e.g., thermal, tactile, and osmotic) come into contact with exposed dentin, the fluid movement in the dentinal tubules stimulates the pulp nerve and causes a brief but sharp pain [3, 4]. Various desensitizing toothpastes have been developed for the relief of this pain, which can be reduced by dentinal tubule occlusion. Most desensitizing toothpastes contain active ingredients, such as calcium carbonate, arginine, and strontium acetate [9, 26]. The desensitizing effect of these toothpastes has been proven in previous studies; however, the durability of the effect is controversial, inasmuch as it is unstable in the presence of saliva or acid [27, 28].

In this study, we compared the dentin sealing effect of CPNE7-DP–containing toothpaste and two commercially available toothpastes and investigated permanent changes, such as tertiary dentin formation. In the SEM analysis, tubule occlusion was observed in all experimental groups but not in the negative control group; however, not all open dentinal tubules were completely occluded. In addition, we observed a little difference in the tubule occlusion between disks brushed for 2 weeks and those brushed for 4 weeks. Because the SEM images depicted only the tooth surface, SEM may be insufficient for the evaluation of the entire dentin sealing effect. We also measured the dentinal fluid flow by microleakage analysis. The dentinal fluid flow was decreased in groups 1, 3, and 4 in comparison with the negative control, but it was still present. This finding may be attributable to the partial occlusion of dentinal tubules observed in the SEM image, suggesting that the pain is partially reduced but still exists. In group 2, in which the CPNE7-DP–containing toothpaste was used, the dentinal fluid flow was significantly decreased in comparison with group 1 as well as the negative control. Of more importance is that we observed newly formed tertiary dentin with tubular structure in group 2 of the dogs’ teeth but no such change in the other groups.

CPNE7-DP, a synthetic oligopeptide derived from CPNE7 protein, was studied in previous research. Lee et al. demonstrated that CPNE7-DP was noncytotoxic, induced odontoblast differentiation in vitro, and induced regeneration of tubular dentin in models with shallow and deep cavities. In addition, they confirmed that peritubular dentin formation was induced by the CPNE7-DP treatment in the model of beagle tooth defects to promote dentinal tubule occlusion, and the volume of the dentinal fluid flow was significantly reduced [23]. These findings—less dentinal fluid flow and newly formed tertiary dentin—were essentially in agreement with the results in group 2 of this study. Therefore, we suggest that the use of toothpaste containing CPNE7-DP can cause permanent changes, such as tertiary dentin formation, which would result in long-term dentin sealing effects.

In the toothpaste used in group 3, calcium carbonate and arginine were active ingredients. The combination of these two substances forms a positive complex with the negatively charged dentin surface, which facilitates tubular occlusion [29]. Other studies have demonstrated that ions of strontium acetate, the active ingredient of the toothpaste used in group 4, were exchanged with calcium ions, which caused the formation of strontium crystals within dentinal tubules; thus, strontium can cause occlusion of dentinal tubules [30–32]. Therefore, we speculate that partial tubule occlusion occurred in groups 3 and 4 as a result of the action of these active ingredients. Several reports have demonstrated that the combination of arginine and calcium carbonate is more effective than strontium acetate in the treatment of dentin hypersensitivity [33–35]. Similarly, we found that microleakage volume was smaller in group 3 than in group 4. In addition, calcium carbonate is able to induce in vitro cell differentiation of human dental pulp stem cells into odontoblasts [36], and strontium at specific doses could influence proliferation, odontogenic differentiation, and mineralization of human dental pulp stem cells in vitro via the calcium-sensing receptor [37]. Nevertheless, in this study, hard tissue formation was not observed in groups 3 and 4, in which toothpaste containing calcium carbonate and strontium acetate, respectively, was used.

Our results indicate that CPNE7-DP–containing toothpaste can induce tertiary dentin formation to promote a sustained dentin sealing effect and act as a successful desensitizer. In addition, the effect was evaluated in both in vitro and in vivo assessments. This method of analysis is useful for precise evaluation and understanding of therapeutic agents for dentin hypersensitivity. A limitation of this study is that we did not evaluate the stability of CPNE7-DP–containing toothpaste in a dietary acid challenge, and this should be evaluated in future studies.

Altogether, our findings have important implications for the use of CPNE7-DP as a novel biological active ingredient in the treatment of dentin hypersensitivity. Ultimately, treatment with CPNE7-DP–containing toothpaste for dentin hypersensitivity may offer a new and fundamental way with a low risk of recurrence and little microleakage. Furthermore, CPNE7-DP has the potential for being used widely in combination with dental materials in clinical practice.

## Conclusions

This study demonstrated that CPNE7-DP–containing toothpaste induced not only a reduction in dental fluid flow but also the biological formation of tertiary dentin. The formation of tertiary dentin was not observed with the use of toothpastes containing calcium carbonate, arginine, or strontium acetate. Thus, CPNE7-DP–containing toothpaste could be a promising agent with long-term dentin sealing effects that could help relieve dentin hypersensitivity.

## Supplementary Information

**Additional file 1: Supplementary Table 1.** Ingredient and content of experimental toothpastes.

**Additional file 2: Supplementary Table 2.** Raw data of microleakage analysis.

## Data Availability

All data generated or analysed during this study are included in this published article and its supplementary information files.

## Abbreviations

CPNE7-DP: CPNE7-derived oligopeptide
SEM: Scanning electron microscopy
EDTA: Ethylenediaminetetraacetic acid
PBS: Phosphate-buffered saline
