# Supplementary material for: Desensitizing toothpastes for dentin sealing and tertiary dentin formation in vitro and in vivo: a comparative analysis
Source: BMC Oral Health. 2022 Nov 11;22:483. doi: 10.1186/s12903-022-02558-8 (PMC9652853; doi:10.1186/s12903-022-02558-8)
Supplement: Supplementary file 1 — Additional file 1: Supplementary Table 1. Ingredient and content of experimental toothpastes. [file 12903_2022_2558_MOESM1_ESM.docx]

Supplementary table 1. Ingredient and content of experimental toothpastes

| Step | component | Ingredient | Content (Wt %) |
| --- | --- | --- | --- |
| 1 | Solvent | Purified water | 21.587 |
|  | Humectant | D-Sorbitol Solution | 30 |
| 2 | Staple | Tricalcium phosphate | 32 |
|  |  | Aminocaproic acid | 0.2 |
|  |  | Allantoin | 2 |
|  | Humectant | Sodium PCA solution | 3 |
|  | Viscosity modifier | Hydrous silicic acid | 4 |
|  | Diluting agent | Hydryxyapatite | 0.05 |
|  | Peptide (Group 2 only) | CPNE7-derived oligopeptide | 0.002 |
|  | Sweetening agent | Enzyme-treated stevia | 0.1 |
|  |  | Xylitol | 0.1 |
| 3 | Humectant | (Concentrated) glycerin | 2 |
|  | Viscosity modifier | Xanthan gum | 0.3 |
| 4 | Humectant | (Concentrated) glycerin | 2 |
|  | Viscosity modifier | CMC (Carboxymethyl Cellulose Sodium Salt) | 0.6 |
| 5 | Surfactant | Cocoylmethyltaurate | 1.2 |
| 6 | Flavoring agent | Green tea extract | 0.01 |
|  |  | Chamomile extract | 0.01 |
|  |  | Rosemary extract | 0.01 |
|  |  | Myrrh tincture | 0.01 |
|  |  | Rhatany tincture | 0.01 |
|  |  | Chamomile tincture | 0.01 |
|  |  | Mastic oil 40 HF-60662 | 0.001 |
|  |  | Propolis extract | 0.05 |
|  |  | Grapefruit seed extract | 0.1 |
|  |  | Spearmint B71228 | 0.05 |
|  |  | Peppermint oil 81689 | 0.6 |
|  | Total |  | 100 |
