# Supplementary material for: Desensitizing toothpastes for dentin sealing and tertiary dentin formation in vitro and in vivo: a comparative analysis
Source: BMC Oral Health. 2022 Nov 11;22:483. doi: 10.1186/s12903-022-02558-8 (PMC9652853; doi:10.1186/s12903-022-02558-8)
Supplement: Supplementary file 2 — Additional file 2: Supplementary Table 2. Raw data of microleakage analysis. [file 12903_2022_2558_MOESM2_ESM.docx]

Supplementary table 2. Raw data of microleakage analysis

| Group | 0 | 90 | 180 | 270 | 360 | 450 | 540 | 630 | 720 | 810 | 900 | 990 | 1020 (sec) |
| --- | --- | --- | --- | --- | --- | --- | --- | --- | --- | --- | --- | --- | --- |
| Negative control | 0 | 408.697 | 499.780 | 553.762 | 593.120 | 641.901 | 3729.700 | 9153.175 | 9225.217 | 9225.217 | 9225.217 | 9225.217 | 9225.217 |
| Group 1 | 0 | 40.045 | 76.361 | 113.461 | 139.602 | 166.920 | 211.611 | 253.816 | 294.777 | 346.175 | 393.451 | 476.780 | 507.697 |
| Group 2 | 0 | 26.844 | 42.744 | 47.701 | 51.234 | 55.209 | 58.645 | 60.853 | 61.393 | 64.534 | 63.699 | 63.748 | 67.331 |
| Group 3 | 0 | 83.853 | 123.342 | 138.621 | 197.347 | 229.017 | 257.382 | 284.210 | 308.158 | 338.781 | 358.084 | 366.656 | 374.704 |
| Group 4 | 0 | 77.097 | 113.314 | 143.250 | 174.118 | 202.974 | 235.560 | 267.950 | 304.363 | 339.452 | 365.216 | 392.109 | 404.034 |
